# Stimuli-Responsive Hydrogels for Oral Probiotic Delivery: Balancing Protection and Site-Specific Release

**DOI:** 10.3390/gels12100861

**Published:** 2026-09-24

**Authors:** Sungwon Jung, Sookyung Noh, Hyeonseung Lee, Sohyeon Park

**Affiliations:** 1 Department of Chemical and Biological Engineering, Hanbat National University, 125 Dongseo-daero, Yuseong-gu, Daejeon 34158, Republic of Korea; skyungn76@gmail.com; 2 School of Food Science and Biotechnology, College of Agriculture and Life Sciences, Kyungpook National University, 80, Daehak-ro, Buk-gu, Daegu 41566, Republic of Korea; dlgustmd77@naver.com

**Keywords:** probiotics, hydrogels, stimuli-responsive delivery, oral delivery, gastrointestinal protection, site-specific release

## Abstract

Successful oral probiotic delivery requires protection of viable cells during gastrointestinal transit followed by efficient release at the target intestinal site. Stimuli-responsive hydrogels can provide both functions, although network properties that restrict acid, bile, and enzyme transport may also delay the structural changes required for release. This review discusses how crosslink density, particle geometry, the internal microenvironment, and interfacial layers shape this protection–release relationship. pH-, enzyme/microbiota-, redox-, and multi-stimuli-responsive systems are compared with emphasis on the structural changes that allow bacterial escape. Because probiotic cells are much larger than the molecular mesh of most hydrogels, release generally requires network opening through swelling, formation or enlargement of cell-scale pores or defects, erosion, fracture, de-crosslinking, or dissolution rather than ordinary molecular diffusion. Delivery performance should therefore be evaluated using release kinetics, viability of released cells, retained cells, and total viable recovery in sequential gastrointestinal models. Strain-specific testing, storage studies, and in vivo validation will also be important. The practical goal is a reproducible protection–release window that maintains cell viability without delaying release.

## 1. Introduction

Probiotics are commonly defined as live microorganisms that confer a health benefit when administered in adequate amounts [1,2,3,4,5,6,7]. Oral delivery exposes them to several forms of stress before they reach the intended site. Dehydration, oxygen, heat, osmotic changes, and interfacial damage may occur during processing and storage; gastric acid, digestive enzymes, bile salts, and osmotic or ionic shifts challenge cell survival during gastrointestinal transit, whereas competition with resident microorganisms can further limit persistence and functional activity after arrival at the target site [8,9,10,11,12].

Encapsulation can reduce direct contact between probiotic cells and adverse environmental conditions. Hydrogels are particularly useful because they form water-rich polymer networks under relatively mild conditions and can be adjusted through polymer composition, crosslinking, particle size, multilayer assembly, and surface modification [13,14,15]. Alginate, chitosan, pectin, starch and cellulose derivatives, carrageenan, gelatin, and whey proteins have all been used as food-derived or widely used biopolymer matrices and coatings [16,17,18,19,20,21,,22,23,24,25,26].

High viable counts after a gastric challenge do not necessarily indicate successful delivery, because a dense carrier may retain most cells instead of releasing them in the intestine. Conversely, rapid swelling may improve release while compromising gastric protection. Encapsulation efficiency and post-digestion viability should therefore be interpreted together with cells recovered from the release medium, cells remaining in the carrier, and the unrecovered fraction [15,27,28,29,30,31,32,33]. Recent reviews have summarized the construction strategies and stimulus classes used for controlled probiotic release [34,35,36,37]. In contrast, the present review centers on the protection–release trade-off and treats structural transition, viable-cell release, and total recovery as coupled design outputs.

Stimuli-responsive hydrogels are intended to control the change from a protected state to a releasable one. Gastrointestinal changes in pH, ionic composition, enzyme activity, microbial metabolism, and local or disease-associated redox conditions can alter polymer charge, crosslink stability, water uptake, erosion, and permeability [38,39,40,41,42]. The trigger is effective only if it reaches the responsive groups and produces a structural change large enough for the cells to leave the matrix (Figure 1).

For oral probiotic delivery, gastric resistance is only the first requirement. A hydrogel must protect the encapsulated cells during gastrointestinal transit and then undergo a timely structural transition at the intended release site. Accordingly, this review links network transport and stimulus-triggered swelling, pore formation, erosion, fracture, or de-crosslinking to viable-cell release kinetics and total viable recovery. Biological variability and experimental conditions that affect these outcomes are also considered. Representative experimental studies are summarized in Table 1, highlighting that direct release measurements and cell recovery remain less commonly reported than gastrointestinal survival.

## 2. Hydrogel Design for Probiotic Protection

### 2.1. Network Transport, Crosslinking, and the Internal Microenvironment

Hydrated hydrogel networks generally remain permeable to water, protons, salts, bile components, and digestive enzymes, particularly through interconnected pores or during ion exchange. Their protective effect depends on how transport resistance, local buffering, polymer–stressor interactions, and water activity delay equilibration with the external medium [13,48,56,57], meaning that acidification may be slowed rather than completely prevented.

Increasing crosslink density reduces chain mobility and water uptake and can slow acid and bile transport, thereby improving short-term protection [58,59,60,61]. If the network becomes too dense, however, nutrient exchange, post-stress recovery, trigger penetration, and de-crosslinking may all be delayed. The practical target is therefore a density that preserves integrity during transit while still allowing release within the intended period. Swelling, erosion, and ion exchange connect the protective phase with release. Ionizable polysaccharides may contract in acidic media and expand after their functional groups become charged at higher pH. Ionic gels can also lose crosslinks when multivalent ions are exchanged, chelated, or displaced by phosphates and other intestinal components [62,63]. Polymer composition, crosslinker identity, curing time, ionic strength, and carrier geometry determine which process dominates. Kim and co-workers developed a nanostructured hydroxyapatite/alginate composite hydrogel by combining ionic alginate gelation with in situ hydroxyapatite formation. Hydroxyapatite neutralized protons entering the beads, allowing the internal pH to be maintained during a 2 h gastric challenge, while the composite subsequently disintegrated at intestinal pH and completely released *L. rhamnosus* GG. Controlling the intraparticle microenvironment therefore offers a route to gastric protection that does not require a denser network [48].

Because bacterial cells (typically 1–3 μm) are much larger than the nanometer-scale mesh size of most hydrogels, their release from intact, uniformly crosslinked networks is unlikely to occur through conventional molecular diffusion. Escape instead generally requires macroscopic expansion, formation or enlargement of cell-scale pores or defects, surface or bulk erosion, fracture, or complete de-crosslinking [56,57,64]. Low cell recovery should not automatically be described as slow diffusion when incomplete network disassembly is a more plausible explanation.

Cell location and physiological state add another layer of variability. Cells close to the particle surface encounter external stress sooner than those in the core, while high local cell densities can change pH, gas accumulation, metabolite levels, and polymer organization. Acid and bile tolerance, surface charge, and metabolic activity also differ among strains [65,66]. Formulation optimization is therefore most informative when carried out with the intended probiotic strain.

### 2.2. Particle Size, Morphology, and Interfacial Layers

Particle size changes both transport distance and interfacial area. Small particles favor mixing, dose uniformity, sensory quality, and recovery, but their high surface-to-volume ratio speeds exchange with the surrounding medium. Large beads provide longer transport paths and may buffer short gastric exposures more effectively, although they can release cells slowly and may not suit every food or dosage format [58,67]. Size effects are difficult to interpret when composition and particle dimensions are changed at the same time.

Carriers with similar mean diameters may nevertheless differ substantially in surface area, pore structure, and defect density. Spherical beads, irregular fragments, microgels, porous matrices, and core–shell particles may have similar nominal sizes but very different surface areas, defects, and transport pathways. Mixing, pumping, drying, or mastication can also introduce cracks that later dominate acid penetration and release. A size distribution and representative microscopy are therefore more useful than a single average value.

Interfacial coatings alter transport at the particle boundary without requiring the entire matrix to be made denser. Chitosan is often deposited on alginate by electrostatic complexation, while proteins and other polysaccharides can form additional shells or multilayers [14,50,68,69,70]. Continuous coating may reduce early contact with acid and bile while leaving a less restrictive core. Its thickness, continuity, charge, and dissolution rate still need control; a shell that does not open merely moves the release barrier to the interface. Anselmo et al. deposited alternating chitosan and alginate layers directly onto individual *Bacillus coagulans* cells using electrostatic layer-by-layer assembly. Two chitosan/alginate bilayers protected the probiotics against simulated gastric fluid and 4% bile salts for up to 2 h and enhanced their intestinal survival in vivo. However, increasing the number of deposited bilayers progressively delayed the onset of bacterial growth, illustrating both the protective benefit and the potential post-release recovery penalty of thicker interfacial coatings [68]. Interpretation of carrier performance also depends on the physiological state of the probiotic. Spore-forming probiotics such as Bacillus coagulans have a substantially higher baseline resistance to gastrointestinal stresses than fragile vegetative cells [71,72]. Therefore, high post-digestion survival of spore-containing formulations should not be interpreted as evidence of hydrogel protection in isolation. Carrier-specific protection is better estimated by comparing encapsulated and unencapsulated spores under the same sequential gastrointestinal conditions, while release, germination, and post-release outgrowth should be evaluated separately. Accordingly, cross-study comparisons should distinguish spore-forming and vegetative probiotics rather than ranking carrier performance solely on final viable counts.

In core–shell and multilayer particles, the outer layer controls when fluid and the trigger reach the inner network, whereas the core determines whether the cells remain viable and can be liberated after the gate opens [73,74,75]. These functions remain coupled: shell dissolution may not overcome a densely crosslinked core, while an overly impermeable shell may prevent the trigger from reaching the inner domain.

### 2.3. Material Selection and Probiotic Compatibility

Alginate remains a common starting material because divalent cations induce rapid gelation under conditions that are usually compatible with living bacteria [57,63,76,77]. Its carboxylate-rich network contracts in acid and swells or loses ionic crosslinks in intestinal media. Alginate gels can nevertheless be porous, heterogeneously crosslinked, and sensitive to ion exchange. Molecular composition, molecular weight, calcium concentration, curing conditions, and test-medium composition all influence the outcome.

Chitosan can reinforce an alginate surface and reduce permeability, but it is not necessarily an inert coating. Its molecular weight, degree of deacetylation, concentration, and charge state affect both film formation and interactions with bacterial surfaces. Depending on the strain and coating conditions, those interactions may reduce viability [67,78,79,80,81]. Cell counts before and immediately after coating should therefore be compared with counts obtained after gastrointestinal exposure.

Pectin, resistant starch, guar gum, cellulose derivatives, and related polysaccharides can reinforce the matrix while providing components that resist upper-gastrointestinal digestion and are susceptible to colonic enzymes or fermentation [17,38,79]. Their degradation rates depend on substitution, branching, crystallinity, and microbiota composition. Claims of microbiota responsiveness are strongest when degradation and release are measured directly in a relevant biological model.

Protein hydrogels and polysaccharide–protein composites broaden the available gelation mechanisms and can alter water retention, interfaces, and mechanical strength [16,44,49]. Proteins may contribute buffering or nutrients, but they can also be degraded by gastric proteases or damaged by heat. The behavior of a composite cannot be assumed from its individual ingredients; it needs to be evaluated as a single network.

Material selection should be matched to the target region, probiotic strain, processing route, storage conditions, acceptable degradation products, and regulatory category (Table 2). For foods and nutraceuticals, a reproducible response based on familiar ingredients may be more practical than an elaborate chemistry that relies on reactive crosslinkers or non-food-grade components.

## 3. Stimuli-Responsive Hydrogels for Site-Specific Probiotic Release

Responsive systems are grouped below by the trigger that opens the network. The triggers differ less in whether they work than in how directly network opening has been demonstrated for live cells (Figure 2, Table 3).

### 3.1. pH-Responsive Hydrogels

The gastrointestinal pH gradient is the most widely used trigger in oral hydrogel systems. Carboxyl-containing polymers are less ionized in acid, which reduces electrostatic repulsion and helps maintain a compact network. Deprotonation at higher pH increases charge repulsion, hydration, and permeability [38,82,83,84]. In ionic gels, the same transition can also accelerate ion exchange and crosslink loss.

Although alginate provides both gastric protection and pH-dependent behavior, its swelling and erosion are also governed by crosslink density, calcium distribution, particle size, ionic strength, phosphate concentration, and exposure time [19,62,85]. Release attributed to pH may therefore reflect a combination of deprotonation and de-crosslinking, and simultaneous measurements of swelling, mass loss, and cell release can help distinguish their contributions. In earlier work, Kim and co-workers incorporated exfoliated bentonite into alginate hydrogels to reduce pore size and delay gastric-fluid penetration. The optimized bentonite/alginate nanocomposite had a pore size approximately half that of alginate alone; following a gastric-pH challenge, the loss of *L. rhamnosus* GG was 1.43 log CFU/g, compared with a 6.25 log CFU/g decrease in alginate beads. The composite remained structurally intact during gastric passage, subsequently disintegrated in the intestine, and produced approximately sixfold higher viable fecal recovery in mice than alginate alone [45]. More recently, Cao et al. fabricated interpenetrating pea-protein-isolate/low-methoxyl-pectin microgels by emulsion-templated Ca^2+^ crosslinking. The optimized formulation achieved 94.80% encapsulation efficiency; the microgels were 43–63 μm in diameter, and encapsulated *Lacticaseibacillus paracasei* YWC-01 remained above 9 log_10_ CFU/g after in vitro digestion, corresponding to less than a 0.4-log reduction compared with a 3.2-log reduction in free cells. Although these results demonstrate strong gastrointestinal protection, direct time-resolved release and complete cell mass balance remained less extensively characterized [86].

Enteric coatings can create a more distinct pH gate. The hydrogel core protects the cells, while the outer material limits fluid entry in the stomach and dissolves or becomes permeable above a selected pH [87,88,89]. This arrangement is well suited to small-intestinal delivery. Colon targeting is less certain because intestinal pH varies with diet, disease, age, transit time, and location, and the dissolution threshold may be reached before the colon. The nominal dissolution pH of a coating does not directly determine when viable cells will appear in the surrounding medium. Release also depends on the onset, rate, and extent of coating dissolution and on the additional time required for the exposed core to swell or disassemble. Sequential tests should report both the first increase in permeability and the time required to recover a defined fraction of viable cells.

### 3.2. Enzyme- and Microbiota-Responsive Hydrogels

Enzyme- and microbiota-responsive systems use polymers or crosslinks that resist human digestive enzymes but are cleaved or fermented by activities enriched in the lower gastrointestinal tract [57,90,91]. Pectin, resistant starch, guar gum, dextran derivatives, and related polysaccharides can function as both structural materials and degradable substrates.

Here the controlling step is biological degradation of the polymer. Backbone cleavage or disruption of enzyme-labile crosslinks can reduce molecular weight, enlarge pores, and promote erosion. To attribute release to a biological trigger, cell recovery measurements should be accompanied by evidence of trigger-dependent polymer degradation, mass loss, rheological change, or release. Incubation time by itself does not establish the mechanism. Although nitroreductase catalysis is a redox process, NTR-responsive systems are grouped here with enzyme/microbiota-responsive platforms because the trigger is enzymatic and microbiota-associated. Chen et al. designed a nitroreductase-labile peptidic supramolecular hydrogel for the oral delivery of *Escherichia coli* Nissle 1917. The hydrogel protected the bacteria against acidic and bile-salt conditions, whereas intestinal nitroreductase cleaved the responsive motif, induced supramolecular disassembly, and triggered local bacterial release. In a murine colitis model, the formulation improved inflammatory and intestinal-barrier outcomes, although the dependence of release on interindividual nitroreductase activity remains to be established [42]. By comparison, Dafe et al. encapsulated *L. plantarum* in extruded pectin/starch hydrogels and examined survival during sequential simulated gastric and intestinal exposure. Free cells were almost completely inactivated, whereas pectin and pectin/starch hydrogels retained 5.15 and 6.67 log CFU/g, respectively. However, because no fecal-community experiment, enzyme-depletion control, or sterile control was included, this study supports lower-gastrointestinal protection and release more strongly than microbiota-specific triggering [27].

Because enzyme abundance and activity vary among individuals and with diet, antibiotics, disease, age, and microbiome composition [92,93,94], results obtained with purified enzymes, single cultures, and fecal communities should not be treated as equivalent. The selected model should instead be matched to the question being addressed: chemical susceptibility, community-dependent degradation, or physiological colon targeting.

Some fermentable carrier components also serve as prebiotic substrates [95]. This can support the delivered probiotic, but it complicates interpretation because fermentation changes local pH, gas production, community composition, and degradation rate. The payload may itself contribute to polymer breakdown or environmental change, creating feedback between the cells and the release trigger.

### 3.3. Redox- and Disease-Associated Responsive Hydrogels

Redox-responsive hydrogels commonly use disulfide or related cleavable bonds to reduce network connectivity under reducing conditions [96,97]. Such cleavage can produce a sharper structural transition than gradual pH-induced swelling and can be combined with another gate. Although the evidence base remains smaller than that for pH-responsive systems, probiotic-specific platforms triggered by pathogen-derived H_2_S, inflammation-associated ROS, as well as platforms incorporating redox-sensitive diselenide bonds, now provide several in vivo proofs of concept. Xiao et al. prepared a self-crosslinked thiolated hyaluronic acid hydrogel containing *L. rhamnosus*. Hydrogen sulfide generated by surrounding intestinal pathogens cleaved the disulfide-crosslinked network, causing local hydrogel degradation and rapid bacterial release. A 4% (*w*/*v*) hydrogel improved probiotic survival under gastrointestinal challenges and produced greater therapeutic benefit than free cells in a *Salmonella*-induced enteritis model, although the trigger is inherently dependent on pathogen-associated H_2_S production [40].

Redox conditions in the gastrointestinal tract vary with oxygen diffusion, diet, inflammation, microbial metabolism, and luminal composition [98,99,100]. In vitro studies often rely on a defined reducing agent chosen for proof of concept. Rapid disassembly under those conditions does not demonstrate colon-specific release unless the concentration and exposure time are physiologically justified.

The synthesis and breakdown chemistry must also be compatible with living cells and the intended product category. Reactive reagents, residual crosslinkers, or cleavage products that are acceptable in a pharmaceutical platform may be unsuitable for food use. Redox responsiveness is therefore better treated as an emerging or secondary trigger until probiotic-specific in vivo evidence and safety data are stronger.

### 3.4. Multi-Stimuli and Sequentially Gated Systems

Multi-stimuli systems combine mechanisms such as an acid-stable shell, an ionically crosslinked core, and a microbiota-degradable component. The value lies in sequence: one layer limits gastric exposure, another begins to swell in the intestine, and a later event accelerates colonic disassembly. This arrangement can reduce dependence on any single physiological variable. Huang et al. constructed a double-layer hydrogel consisting of a carboxymethyl-cellulose supramolecular inner layer and a dialdehyde-alginate-crosslinked carboxymethyl-chitosan outer layer. No probiotic release was detected during 2 h in simulated gastric fluid, whereas release began following transfer to simulated intestinal fluid and reached 90% after 6 h. The formulation also markedly increased intestinal retention and oral bioavailability in animal models, but the contributions of sequential network opening and mucoadhesion were not fully separated [50]. This study illustrates how a gastric barrier and a delayed intestinal-opening mechanism can be placed in separate structural domains, while also showing that release and mucosal retention must be evaluated as distinct outcomes. Tang et al. combined a gastric pH-responsive alginate/thioketal hydrogel with an EGCG–Mn nanoparticle coating on *L. rhamnosus* GG. The bacterial coating increased oxidative-stress resistance by 6.28-fold, while the dual-responsive hydrogel provided a 71.6% survival rate under gastric challenge and promoted bacterial release and retention at colitic lesions. This platform provides a recent example of pH/ROS sequential gating, although its multiple protective, antioxidant, targeting, and therapeutic components make mechanism-specific attribution difficult [101].

Although sequential gating can reduce dependence on a single physiological variable, each additional polymer, coating step, and crosslinking reaction may increase batch variability, processing stress, storage instability, and analytical burden. The role of each component should be stated as a gate, trigger, structural support, or release amplifier, and its contribution should be demonstrated by an appropriate control. Living–synthetic hybrid hydrogels extend the same logic by making the delivered cells part of the responsive material itself [102].

Separate gastric and intestinal endpoint tests may overlook structural changes carried from one phase into the next. Because acid exposure can alter the network subsequently exposed to intestinal pH or enzymes, formulations should be evaluated by sequential media transfer using realistic residence times and dynamic pH or ionic conditions.

## 4. Integrating Protection and Responsive Release

### 4.1. The Protection–Release Window and Common Failure Modes

Protection and release are often reported as separate formulation properties even though both arise from the same structure. Increasing crosslink density, coating thickness, or hydrophobic association may slow gastric stressors but also delay hydration, trigger penetration, erosion, and cell escape. The number of viable cells delivered to the target therefore shows a non-monotonic relationship with barrier strength: both an under-protective and an over-protective carrier can perform poorly.

Premature leakage exposes cells before gastric transit is complete. Protected-but-unreleased cells remain viable inside a carrier that never opens. Some cells reach the medium only after losing culturability or metabolic competence. And when only one compartment is counted, incomplete sampling can manufacture an apparent benefit. These failure modes are routinely conflated in the literature, and each calls for a different formulation change. To translate these failure modes into practical formulation decisions, Table 4 summarizes their major structural causes, corresponding design strategies, and the measurements needed to verify whether the intended protection–release balance has been achieved.

### 4.2. Structural Strategies for Coupled Control

Core–shell particles provide the clearest physical separation of roles. The shell can serve as a temporary gastric gate, while the core is adjusted for cell compatibility and later disassembly. Layer-by-layer coatings allow finer control of thickness and composition, although defects or incomplete coverage may dominate the result. Direct characterization of coating quality is more reliable than inferring it from improved survival.

Interpenetrating and double networks pair a mechanically stable scaffold with a degradable or ion-responsive network. The first preserves particle integrity during processing, while the second controls swelling or creates release pathways. Sacrificial components can generate cell-scale pores after dissolution or microbial degradation. These strategies are most convincing when pore formation is measured and related to bacterial dimensions.

The required structural transition and validation strategy depend on the intended gastrointestinal target (Table 5). A formulation should include only components that provide a defined function, remain compatible with the intended strain, and can be produced reproducibly; adding poorly characterized transitions narrows the operating window and complicates manufacturing without improving the required sequence.

### 4.3. A Minimum Evaluation Framework

Before gastrointestinal testing, the formulation should be characterized for initial loading, process survival, cell distribution, particle-size distribution, swelling, and mechanical or rheological properties. Encapsulation efficiency alone cannot show whether the process injured the cells or whether the dose can be reproduced.

Sequential exposure with fully specified media is the informative case, and the specification has to include agitation and the transfer procedure, which are omitted far more often than pH or enzyme content [103,104]. Samples are needed during the protective phase and after the expected trigger. Static endpoint tests are useful for screening, whereas dynamic or semi-dynamic models are preferable when gradual pH change, dilution, mixing, or ionic shifts influence the response.

Protection and release measured in separate experiments cannot be reconciled afterwards. Within a single sequential run, viable cells in the external medium and those remaining in the carrier give total viable recovery at each stage. This CFU-based quantity should not be conflated with physical release fraction, because loss of culturability after release can lower viable counts without indicating retention in the matrix. Accordingly, viable release yield, viable retained fraction, released-cell viability, and time to a defined release level should be reported separately; when a physical cell balance is claimed, CFU measurements should be complemented by a suitable total-cell or strain-specific assay. Direct numerical comparison across hydrogel systems remains difficult because studies differ in probiotic strain, gastrointestinal media, residence time, initial loading, and the definitions used for survival and release. Quantitative values are therefore most informative when reported together with their experimental conditions rather than interpreted as universal performance rankings. Banerjee et al. optimized 24 μm synbiotic alginate microcapsules containing *L. casei* and explicitly modeled bacterial release across a staged in vitro gastrointestinal system. The microcapsules achieved 98% encapsulation efficiency, and viable cells appeared after approximately 50 min in simulated large-intestinal fluid, with the concentration profile described using a burst-release model. Release time and released-cell concentration were primary outputs here, which remains uncommon [28]. Total counts, membrane-integrity assays, and metabolic measurements can complement colony-forming units, but they do not measure the same biological state.

Cell-release data are more informative when interpreted together with swelling, mass loss, crosslinker release, particle integrity, pore formation, and coating dissolution. These measurements can identify whether cells appear in the medium because the network has opened and can also explain low recovery when structural disassembly is incomplete, allowing more meaningful comparison across formulations (Figure 3).

In vivo studies should distinguish delivery from colonization and therapeutic effect by measuring recovery of the administered strain at the intended region, local viability, residence time, and carrier fate. Fecal counts and downstream health outcomes are also influenced by growth, washout, host responses, and changes in the resident microbiota and therefore cannot by themselves confirm site-specific delivery. Protection, structural response, release, and cell recovery should be assessed within a common sequential framework (Table 6).

## 5. Challenges and Perspectives

### 5.1. Standardized Gastrointestinal Models and Reporting

The field still lacks sufficiently consistent protocols for direct comparison. Simulated gastric and intestinal media vary in pH, buffer capacity, ions, enzyme activity, bile concentration, residence time, and agitation [103,104]. These conditions directly affect swelling, ion exchange, erosion, and trigger activation. Recognized protocols should be used where possible, and departures should be justified in enough detail to reproduce the experiment. Log survival, viable release yield, released-cell viability, total viable recovery, and response time would provide a useful common set of outcomes.

### 5.2. Biological Variability and In Vivo Relevance

Probiotic strains differ in stress tolerance, surface chemistry, growth state, and interactions with polymers, while host pH, transit time, diet, disease, medication, and microbiota composition also vary [105,106]. Microbiota-responsive formulations are especially sensitive to this variation. Testing across more than one relevant biological condition, identifying the activity responsible for degradation, and adding in vivo validation are important when site-specific delivery is claimed. Small-intestinal release, colonic release, mucosal residence, and long-term colonization should not be treated as equivalent targets.

### 5.3. Manufacturing Route, Drying, and Storage Stability

Most responsive hydrogels are first tested as freshly prepared wet particles, whereas commercial probiotics must tolerate processing and storage. The manufacturing route is itself a formulation variable because it determines particle size and size distribution, interfacial area, loading uniformity, and the mechanical or physicochemical stresses experienced by the cells. Extrusion or dripping generally provides mild aqueous processing but tends to produce relatively large particles and may be limited in throughput. Emulsification can generate smaller and more uniform microgels, although additional interfaces, surfactants, shear, and separation steps may affect cell viability and network properties. Electrospraying offers finer control over particle dimensions, but feed rheology, conductivity, applied voltage, and nozzle conditions must be optimized to preserve cell viability and process reproducibility [107]. Three-dimensional printing provides precise control over core–shell or multilayer architectures and can spatially separate protective and release functions; however, printing resolution, extrusion-induced stress, and post-print stabilization can limit viable loading and scale-up. Manufacturing methods should therefore be compared not only by encapsulation efficiency, but also by process survival, particle uniformity, retained stimulus responsiveness, and production throughput [108]. Extrusion, emulsification, pumping, freezing, freeze-drying, spray-drying, and rehydration can change both cell physiology and network structure [109,110,111]. Long-term storage should be treated as a coupled cell–matrix stability problem rather than as cell viability alone. Moisture uptake can increase molecular mobility and accelerate viability loss, while oxygen exposure is particularly important for oxygen-sensitive strains; storage temperature can additionally affect both cellular stability and polymer relaxation. Importantly, a formulation may retain a high viable count while losing the swelling, erosion, de-crosslinking, or coating-dissolution behavior required for site-specific release. Shelf-life studies should therefore report viable counts together with moisture content or water activity, relevant oxygen exposure, and post-rehydration release behavior at multiple storage time points. Oxygen exposure during encapsulation and drying is a further constraint for extremely oxygen-sensitive next-generation strains [112]. This challenge becomes more pronounced for next-generation probiotics, including extremely oxygen-sensitive anaerobes and defined microbial consortia [113]. Oxygen exposure during mixing, encapsulation, drying, storage, and rehydration may reduce viability before gastrointestinal protection is even evaluated. In multi-strain consortia, total CFU can also obscure selective loss or differential release of individual members. Future formulations should therefore consider oxygen-controlled processing and packaging where necessary and quantify strain- or member-specific recovery and release rather than relying solely on total viable counts.

### 5.4. Scale-Up, Safety, Cost, and Regulatory Fit

Acceptable carrier complexity depends on whether the product is a food, dietary supplement, medical food, or pharmaceutical. Food-oriented systems favor familiar ingredients, mild processing, low residual crosslinker, simple quality control, and high-throughput manufacture. Pharmaceutical systems may use more elaborate chemistries but require detailed characterization of residual reagents, degradation products, batch reproducibility, and in vivo safety. A responsive mechanism must fit the intended regulatory pathway as well as the desired release profile.

Translation also depends on batch-to-batch reproducibility, manufacturing cost, ingredient status, residual crosslinkers, degradation products, and practical quality-control requirements. Food and dietary-supplement applications generally favor scalable processes based on food-compatible materials and relatively simple quality attributes, whereas pharmaceutical products may justify more complex responsive chemistries but require more extensive characterization of identity, purity, safety, and reproducibility. The cost and throughput of coating, purification, drying, and controlled-atmosphere processing should therefore be considered alongside release performance. In practice, the most sophisticated responsive system is not necessarily the most translatable; the preferred design is the simplest formulation that reproducibly meets the required protection, release, storage, and safety criteria for the intended product category [114].

### 5.5. From Formulation Screening to Design Rules

Many studies change several formulation variables together and identify a locally optimized composition. This establishes feasibility but yields few transferable design rules. Few studies vary crosslink density, coating thickness, particle size, and cell loading independently while also measuring transport, which is one reason design rules have been slow to emerge. Mechanistic datasets of this kind could support prediction and reduce trial-and-error development. Table 6 sets out what to measure at each stage; Table 7 sets out what counts as evidence for a specific claimed trigger. Transferable design rules require both.

## 6. Conclusions

Stimuli-responsive hydrogels can improve oral probiotic delivery by combining gastrointestinal protection with site-specific release. These functions, however, are governed by many of the same structural properties. Crosslink density, particle size, interfacial layers, ion exchange, and the local microenvironment influence both stressor transport and the subsequent opening or disassembly of the network. Excessive barrier strength may therefore improve gastric protection while delaying cell release.

pH- and ion-responsive systems remain the most established and readily implementable approaches, although their performance depends strongly on gastrointestinal and test-medium conditions. Enzyme- and microbiota-responsive materials offer greater potential for colonic delivery but are affected by biological variability. Redox- and disease-responsive systems remain less established, while multi-stimuli systems can provide better sequential control at the cost of greater formulation complexity. Future studies should evaluate protection and release within the same sequential experiment and distinguish released, retained, and unrecovered cells. Structural changes such as swelling, erosion, de-crosslinking, and particle disintegration should also be measured alongside viable-cell recovery. Storage stability and in vivo target-site recovery remain decisive for commercial development. The formulations that will translate are the ones whose release step is measured as carefully as their gastric survival.

## Figures and Tables

**Figure 1:**
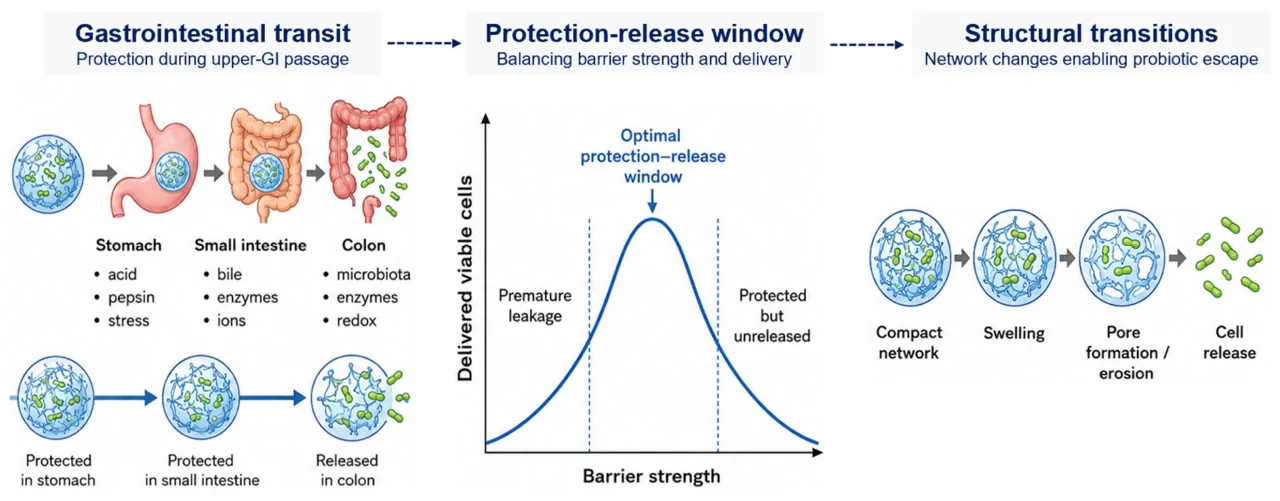
Theprotection–release window for oral probiotic delivery. Increasing barrier strength raises gastric survival but delays trigger penetration and network opening, so the number of viable cells delivered to the target site is non-monotonic in barrier strength.

**Figure 2:**
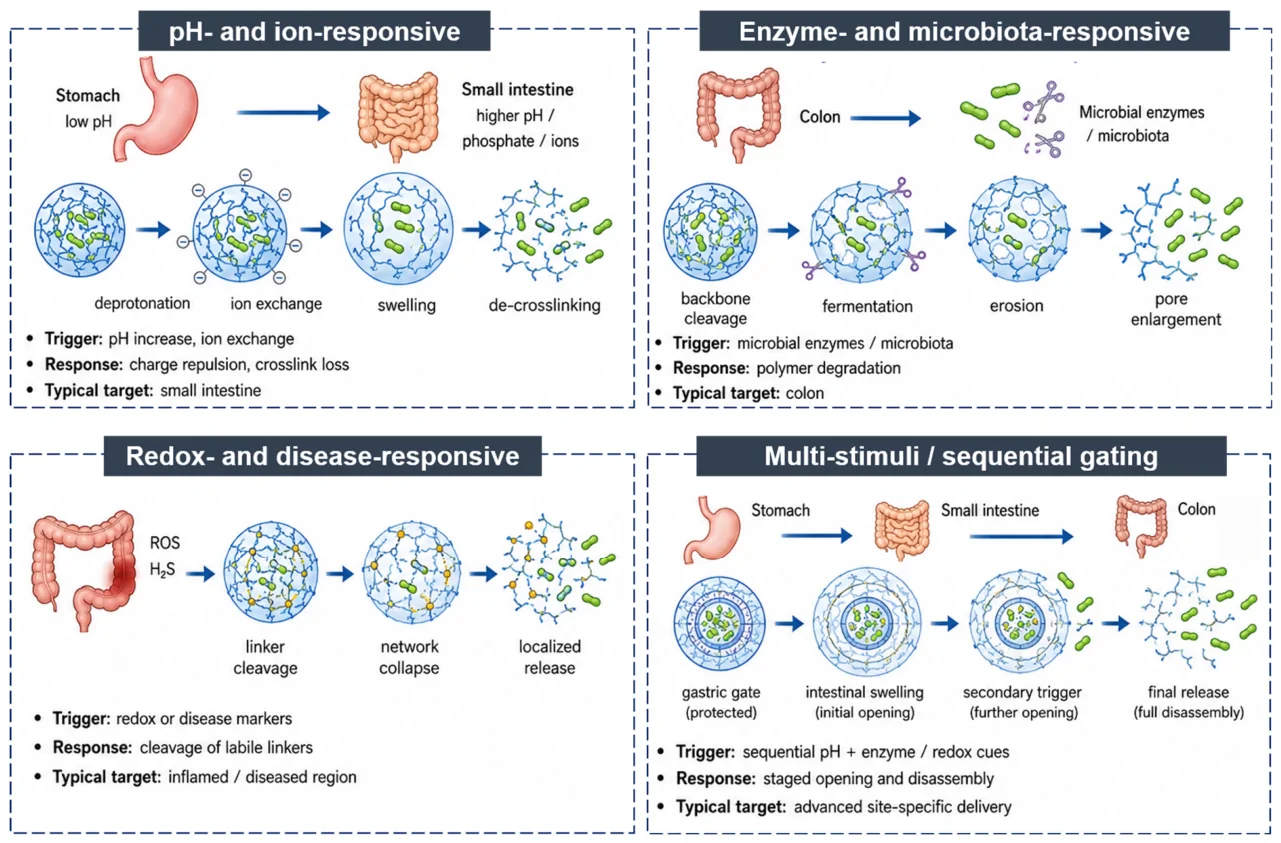
Stimulus-responsive mechanisms and corresponding hydrogel structural changes. The pathways are schematic; not all indicated events occur in every system or in a fixed sequence.

**Figure 3:**
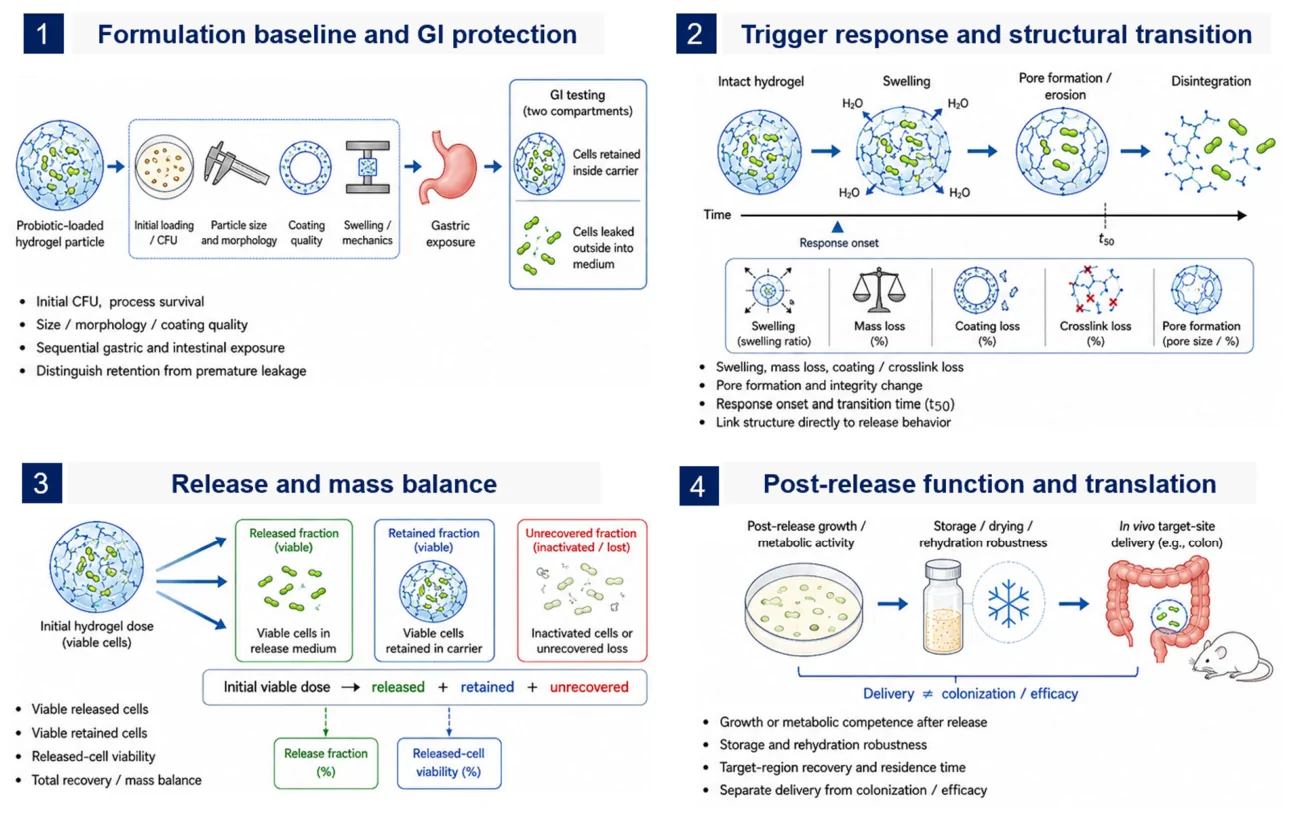
Integrateddesign and evaluation framework for protection, release, and total viable recovery.

**Table 1:** Representativeexperimental studies of hydrogel-based oral probiotic delivery.

Probiotic /Carrier	Responsive or Release Basis	Key Quantitative Outcome	Main Observation /Evidence Gap	Ref.
*L. acidophilus*; alginate or carrageenan beads	pH/ion-mediated swelling and bead disassembly	Encapsulated cells showed ~3-log reduction versus ~7-log reduction for free cells during simulated GI exposure; alginate encapsulation efficiency was 98%.	Yogurt and simulated-GI survival improved, although release was assessed mainly at endpoints, limiting evaluation of the protection–release window.	[43]
*L. rhamnosus*; thiolated hyaluronic acid hydrogel	Pathogen-derived H_2_S cleaves disulfide crosslinks	A 4% (*w*/*v*) hydrogel provided the selected stability; a directly comparable release fraction or release time was not reported.	Localized release improved enteritis outcomes; trigger depends on pathogen H_2_S	[40]
*B. longum*; fish gelatin/sodium alginate double network	Thermal protein gel plus Ca^2+^-crosslinked alginate	Encapsulation efficiency increased from 15.85% to 91.91% as fish gelatin increased to 50 g/L; viability in simulated gastric fluid was >15% versus 1.7% for free cells.	Acid, bile, and heat tolerance increased, but the contribution of the release mechanism was not isolated	[44]
*L. rhamnosus* GG; bentonite/alginate nanocomposite	Gastric-pH resistance; intestinal on-demand disintegration	Gastric challenge caused a 1.43-log CFU/g loss versus 6.25-log CFU/g for alginate alone; viable fecal recovery was ~6-fold higher.	Clay reinforcement increased gastric survival and intestinal delivery; evidence remains largely in vitro	[45]
*E. coli* Nissle 1917; adhesive hydrogel microspheres	Colon-targeted retention and mucosal adhesion	A directly comparable probiotic release fraction was not reported; in vivo colon targeting and retention were emphasized.	Greater colonic residence, immune regulation, and microbiota recovery were observed in colitis mice; retention was emphasized over direct release kinetics	[46]
Probiotic cells; methacrylated HA/thioketal hydrogel	Inflammation-associated ROS cleaves thioketal linkers	Survival increased significantly under simulated digestive conditions, but a directly comparable numerical release fraction was not reported.	Cells were protected and delivery to the inflamed colon increased; activation depends on disease-associated ROS	[41]
*B. coagulans* spores + folate; gellan/kappa-carrageenan/chitosan	pH-dependent swelling and release	Spore release was 28.42% at acidic pH and increased at neutral/alkaline pH.	Intestinal release increased, but the inherent GI resistance of B. coagulans spores limits attribution of survival solely to hydrogel protection.	[47]
*L. rhamnosus* GG; hydroxyapatite/alginate composite	Internal pH buffering plus intestinal alginate disintegration	After 2 h at pH 2, viable counts were 9.19 log CFU/g in the optimized composite versus 3.55 log CFU/g in alginate; complete release occurred at intestinal pH within the 4-h test.	The internal pH was maintained and intestinal release improved; inorganic loading may complicate translation	[48]
*E. coli* Nissle 1917; NTR-labile peptidic hydrogel	Intestinal nitroreductase triggers supramolecular disassembly	NTR-dependent hydrogel disassembly and improved GI viability were demonstrated; a directly comparable release fraction/time was not reported.	NTR-triggered release improved colitis outcomes; interindividual NTR variability remains to be established	[42]
Probiotics; dextran/sulfhydryl-BSA self-healing hydrogel	Self-healing network with prolonged gastrointestinal retention	In vivo probiotic abundance was 35-fold, 8-fold, and 203-fold higher in the intestine, colon, and cecum, respectively, than for free probiotics.	Stress tolerance and intestinal abundance increased, but site-specific release was inferred rather than directly quantified	[49]
Probiotics; CMCL inner layer/DAA-CMCS outer layer	Double-layer gastric barrier followed by intestine-targeted opening	No release occurred during 2 h in SGF; release reached 90% after 6 h. In vivo bioavailability and mucoadhesion were 100.1-fold and 10.6-fold higher than free probiotics at 48 h.	Survival and intestinal delivery improved; the effects of the individual components were not separated	[50]
*L. kefiranofaciens*; pectin/alginate + montmorillonite	Reinforced gastric barrier; colon-oriented disassembly	After 2 weeks of storage, the maximum viability loss among filler groups was 1.21 log CFU/g, whereas PAMT3 freeze-dried microbeads lost only 0.46 log CFU/g.	Viability and colonic colonization improved, but the contribution of each trigger remained unresolved	[51]
*L. reuteri*; HA hydrogel crosslinked with selenocystamine	Redox cleavage of selenocystamine crosslinks	The hydrogel responded to 10 mM GSH or 100 micromolar H_2_O_2_; probiotic survival increased in simulated gastric and intestinal fluids, although a common release metric was not reported.	Delivery and colitis outcomes improved; the physiological relevance of the redox window needs further validation	[52]
*L. plantarum* + resistant-starch nanoparticles; core–shell alginate	Dense prebiotic shell with intestinal de-crosslinking	Probiotic loading was ~10^8^ CFU per bead; 52% viability was retained after 30 days of storage.	Acid protection, storage survival, and colitis efficacy improved; release and post-release growth were not separated	[53]
Probiotics; alginate/low-methoxyl pectin beads	pH/ion response plus microbiota-accessible pectin	After 4 h under simulated intestinal conditions, encapsulated probiotic viability remained at approximately 10^7^ CFU/g.	Colon-targeted delivery and microbiota modulation were enhanced; performance may remain host-dependent	[54]
10-strain probiotic blend; starch core/alginate-pectin shell	pH-sensitive 3D-printed core–shell structure	After 33 days at 4 C, survival was 64.37%. Retention during simulated digestion was 97.22% (oral), 83.33% (gastric), and 82.70% (intestinal), versus 93.62%, 10.92%, and 6.30% for free probiotics.	Storage stability and sequential-digestion retention were high, whereas time-resolved release data were limited	[55]

Note: Quantitative outcomes were added only when a numerical metric was available from the cited study; non-comparable or unreported metrics are identified explicitly to avoid direct ranking across heterogeneous experimental conditions.

**Table 2:** Design variables linking gastrointestinal protection and probiotic release.

Design Variable	Delivery Benefit	Release Risk	Minimum Characterization
Crosslink density	Lower swelling; slower acid/bile transport; stronger matrix	Delayed trigger penetration, pore formation, and de-crosslinking	Crosslinker content; swelling; modulus; ion exchange; release profile
Particle size/morphology	Longer transport path; lower surface-to-volume ratio	Slow or incomplete release; dosage-format constraints	Full size distribution; microscopy; shape and porosity
Surface coating	Boundary barrier with a less restrictive core	Persistent or defective shell; variable protection and release	Thickness; continuity; permeability; surface charge; dissolution or erosion
Polymer degradability	Integrity during upper-GI transit	Slow or host-dependent target-site breakdown	Mass loss; molecular-weight change; enzyme/microbiota dependence
Ionic environment	Stabilization of ionically crosslinked networks	Premature or uncontrolled de-crosslinking	Medium composition; ion concentrations; crosslinker release
Cell loading/distribution	Higher payload and delivered dose	Crowding; local pH/metabolites; biased recovery	Encapsulation efficiency; process survival; spatial distribution; total recovery

**Table 3:** Comparisonof stimulus-responsive strategies for probiotic hydrogel delivery.

Trigger	Structural Response	Main Strength	Main Limitation/Controls
pH and ions	Deprotonation; swelling; ion exchange; enteric dissolution	Simple, established, and compatible with food-grade polymers	Variable GI pH and media; premature release; report onset and response time
Enzyme/microbiota	Backbone cleavage; fermentation; erosion; labile-crosslink cleavage	Stronger colon rationale; possible prebiotic function	Host/microbiome variability; verify degradation in a biological model
Redox	Cleavage of disulfide or other redox-labile links	Potentially sharper network failure; compatible with secondary gating	Limited probiotic and in vivo evidence; justify reductant conditions and safety
Multi-stimuli sequence	Protective gate followed by swelling, degradation, or secondary failure	Staged control and reduced dependence on one trigger	Manufacturing and analytical complexity; demonstrate each component’s function

**Table 4:** Common failure modes in the protection–release window and concise formulation strategies.

Observed Failure Mode	Likely Cause	Formulation Strategy	Key Validation
Premature gastric release	Low crosslink density; rapid swelling; porous or defective coating.	Increase barrier strength; optimize coating integrity and particle size.	Gastric leakage; swelling; coating integrity.
Protected-but-unreleased/delayed release	Dense network; thick shell; slow trigger penetration or disassembly.	Reduce crosslinking or shell thickness; use trigger-labile links or degradable components.	Release onset; t50; retained cells; mass loss.
Loss of viability after release	Harsh processing; reactive chemistry; pH/osmotic stress; prolonged confinement.	Use mild, cell-compatible processing and optimize conditions for the target strain.	Process loss; released-cell viability; post-release growth/function.
Heterogeneous or incomplete release	Broad size distribution; uneven crosslinking/coating; nonuniform cell loading.	Improve size, crosslinking, coating, and loading uniformity.	Size distribution; microscopy; release variability; cell balance.
Host-dependent release	Variation in GI pH, enzymes, microbiota, ions, or disease-associated triggers.	Use a broader response window or complementary sequential triggers.	Release across relevant trigger ranges and sequential GI conditions.

**Table 5:** Design considerations according to the intended gastrointestinal target.

Target/Objective	Required Carrier Response	Design and Key Validation
Small intestine	Remain intact in the stomach; open promptly after the gastric-to-intestinal pH/ion shift.	Use a pH/ion-responsive core or enteric shell. Report leakage, response onset, time to 50% release, and released-cell viability in sequential media.
Colon	Resist upper-GI transit; degrade under colonic enzyme or microbiota activity.	Combine a gastric gate with a degradable polysaccharide. Confirm trigger-dependent degradation/release and target-region recovery.
Disease-associated site	Respond to ROS, H_2_S, nitroreductase, or redox change at disease-relevant levels.	Place a cleavable linker behind a gastric gate. Compare healthy and disease conditions; assess trigger range and product safety.
Mucosal retention	Release cells or retain the carrier near the target mucosa.	Add an adhesive interface without blocking release. Measure target recovery, residence time, and carrier fate; separate retention from colonization.

**Table 6:** Stage-by-stage evaluation of protection, release, and total viable recovery.

Evaluation Stage	Minimum Dataset	Primary Output
Formulation baseline	Initial CFU; viable loading and process survival; size distribution, morphology, swelling/mechanics, and coating quality.	Viable dose, process log loss, and structural variability.
Sequential GI protection	Fully defined media and transfer conditions; viable cells outside and inside the carrier during gastric exposure.	Gastric survival separated from premature leakage and retention.
Trigger response	Time-resolved evidence that the network has opened (see for the evidence appropriate to each claimed trigger).	Response onset and structural-transition time.
Release and viable-cell balance	Viable released cells, viable retained cells, released-cell viability, and unrecovered fraction; add a total-cell or strain-specific assay when physical mass balance is claimed.	Viable release yield, viable retained fraction, t_50_ when applicable, and total viable recovery.
Post-release function	Strain identity plus growth, metabolic activity, or another strain-relevant functional assay.	Biological competence of the released population.
Translation and in vivo	Storage/rehydration survival and retained response; target-region recovery, residence time, and carrier fate.	Product robustness and site-specific delivery, distinct from colonization or efficacy.

**Table 7:** Minimum evidence and controls for validating proposed responsive-release mechanisms.

Claimed Mechanism	Minimum Direct Evidence	Critical Control and Interpretation
pH, ion, or enteric gating	Swelling/size change, mass or crosslinker loss, and coating dissolution/permeability over time.	Match pH and ionic strength; include an uncoated core and sequential exposure. Nominal dissolution pH is not release time.
Enzyme or microbiota degradation	Polymer degradation, rheology or mass loss, and trigger-dependent viable-cell release.	Use no-enzyme, inactive, or sterile controls and a relevant community. Time-dependent erosion alone is not colon-specific.
Redox or disease-associated cleavage	Bond/linker cleavage, network disassembly, trigger dose-response, and viable-cell release.	Use physiological trigger levels, healthy/disease conditions, and a non-cleavable control. Proof-of-concept reagent levels do not show in vivo selectivity.
Pore formation, erosion, fracture, or de-crosslinking	Microscopy or particle-integrity data showing network opening, correlated with cell release.	Remove the degradable component or preserve network integrity. Avoid describing escape through an intact mesh as simple diffusion.
Multi-stimuli sequence	Layer-specific, time-resolved activation in the proposed order, linked to release.	Omit each component and compare isolated versus sequential triggers. Assign a defined role to each component.

## Data Availability

No new data were created or analyzed in this study. Data sharing is not applicable to this article.
